# Effect of Various Acaricides on Hatchability of Eggs of *Rhipicephalus (Boophilus) microplus*

**DOI:** 10.1155/2014/425423

**Published:** 2014-06-26

**Authors:** M. Haque, Jyoti, N. K. Singh, S. S. Rath

**Affiliations:** ^1^Department of Veterinary Parasitology, College of Veterinary Science, Guru Angad Dev Veterinary and Animal Sciences University, Ludhiana 141004, India; ^2^Department of Veterinary Parasitology, College of Veterinary Science & A. H., Mhow, Madhya Pradesh 453 446, India

## Abstract

The effect of commonly used acaricides (amitraz, cypermethrin, deltamethrin, fenvalerate, and flumethrin) on the eggs of *Rhipicephalus (Boophilus) microplus* collected from Ludhiana, Punjab, was evaluated by egg hatch assay (EHA). The regression graph of probit hatchability and per cent inhibition of hatching (IH%) of eggs was plotted against log values of concentration of various acaricides. All concentrations of flumethrin and amitraz caused complete inhibition of hatching, whereas a hatchability of 31.0 ± 6.1, 40.0 ± 5.2 and 19.3 ± 1.7% was only recorded at the highest concentration of cypermethrin, deltamethrin, and fenvalerate, respectively. An increase in the concentration of the acaricide showed a significant effect on the IH% of eggs for cypermethrin (*P* < 0.01) and deltamethrin (*P* < 0.05) but was nonsignificant for fenvalerate. The slope of the regression curve of IH% was utilized for the calculation of the dose of various acaricides causing inhibition of hatching for 95% eggs (LC_95_) and the discriminating dose (DD). Results indicated that maximum DD was recorded for fenvalerate (2.136%), followed by cypermethrin (0.214%) and deltamethrin (0.118%). The results of the current study will be helpful in formulating effective control strategies against ticks.

## 1. Introduction

*Rhipicephalus (Boophilus) microplus* is a widely prevalent tick and assumes great significance in a tropical country like India, where the warm, humid climate favours its perpetuation and propagation. Besides avid blood suckers, these tick species act as the vector of bovine babesiosis and anaplasmosis and also cause 20–30% reduction in the cost of leather due to tick bite marks [1]. Control of ticks is focused on large scale repeated use of acaricides, namely, organophosphates (OP), synthetic pyrethroids (SP), amidines, and macrocyclic lactones, with limited success [2]. Repeated application of these chemicals leads to the development of resistance in the ticks which is considered as the main hindrance for successful pest and vector control program in livestock globally [3].

Along with the use on infested animals these chemical acaricides are also applied in the shed as spray for the elimination of preparasitic free living stages particularly the eggs and the unfed larval stages for complete tick control particularly single host tick* R. (B.) microplus*. This often leads to serious drawbacks, including environmental contamination and even contamination of milk and meat products with insecticide residues [4]. Currently, tick control is more difficult due to the presence of resistant populations to major families of acaricides [5]. After the pioneer report of *γ*BHC resistance in* R. (B.) microplus* recently, Kumar et al. [6] and Sharma et al. [2] reported various populations of* R. (B.) microplus* resistant to diazinon and SP (cypermethrin and deltamethrin), respectively, in India. Further, there is information on ticks double-resistant to OP (diazinon) and SP (deltamethrin and cypermethrin) from Haryana state [7]. Although larval packet test (LPT), originally described by Stone and Haydock [8], has been recommended by FAO as standard bioassay for testing resistance to acaricides in cattle tick* R. *(*B.*)* microplus,* other tests including the larval immersion test (LIT) of Shaw [9] and adult immersion tests (AIT) described by Drummond et al. [10] have been used. However, reports on effects of various acaricides on the egg stage of* R. (B.) microplus* to assess its tick control potential are not readily available. Thus, the current study was undertaken to assess the comparative efficacy of various commercially available acaricides against the eggs of* R. (B.) microplus*.

## 2. Materials and Methods

### 2.1. Collection of Ticks

Live engorged adult female* R. (B.) microplus* ticks were collected from dairy sheds at Haibowal Dairy Complex, Ludhiana, Punjab. Also data related to frequency, type, and mode of acaricide treatment adopted by the owners and their experiences about the commonly used acaricides efficacy were recorded. The ticks were collected in vials, closed with muslin cloth to allow air and moisture exchange, brought to the Entomology Laboratory, Department of Veterinary Parasitology, GADVASU, Ludhiana, cleaned, labeled, and kept at 28 ± 1°C and 85 ± 5% relative humidity for laying of eggs [2].

### 2.2. Acaricide

The commercial products, namely, Cyperkill (10% EC cypermethrin), Butox (1.25% EC deltamethrin), Ticomax (20% EC fenvalerate), Bayticol (1% flumethrin), and Taktic (12.5% amitraz), were used as the stock solution of acaricides. For the experimental bioassay, various concentrations of cypermethrin (100, 200, 300, 400, and 500 ppm), deltamethrin (25, 50, 75, 100, and 125 ppm), fenvalerate (100, 200, 300, 400, and 500 ppm), flumethrin (20, 40, 60, 80, and 100 ppm), and amitraz (250, 500, 750, 1000, and 1250 ppm) were prepared in distilled water from the stock solution and tested against the eggs of* R. (B.) microplus*.

### 2.3. Egg Hatch Assay (EHA)

Egg hatch assay was conducted according to the method of Ribeiroet al. [11] with minor modifications. Approximately 100* R. (B.) microplus* embryonated eggs were placed in a glass tube and immersed for 5 min in 1 mL of the test solution. Subsequently, the solution was decanted and after evaporation of the solvent the tube was covered with a muslin cloth. Eggs were incubated at 28 ± 1°C and 85 ± 5% relative humidity for 14 days, until hatching was completed. Water was used as control and each treatment contained three replicates. The following parameters were compared:

- hatching (%): the number of hatched larvae divided by the total number of incubated eggs;
- percentage inhibition of hatching (IH%) = [(Hatching % control − Hatching % treated)/Hatching % control × 100].

Dose response data were analyzed by probit method [12] using GraphPad Prism 4 software. The concentrations for causing 95% inhibition of hatching (LC_95_) of various acaricides were determined by applying regression equation analysis to the data of IH% and discriminating dose (DD) was determined as 2× LC_95_ [13].

## 3. Results and Discussion

One host tick* R. *(*B.*)* microplus* is one of the most important ixodid ticks infesting dairy animals of India particularly Punjab state [14]. The direct application of acaricides to host animals is the most widely used method for the control of ticks in the region. Spray and injection were mainly used for application of insecticides, while “pour-on” was reported by lesser number of the farm owners. Further, in this region, currently the use of synthetic pyrethroids (cypermethrin, deltamethrin, and fenvalerate) is maximum, whereas the use of formamidine (amitraz) for tick control has started in recent past and was restricted in some farms.

**Figure 1:**
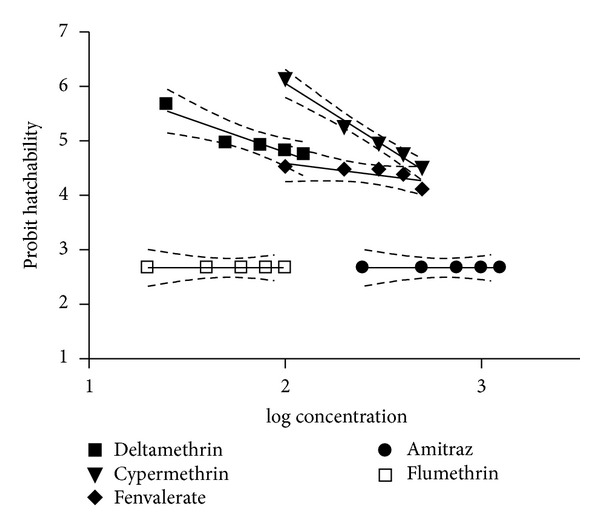
Dose-probit hatchability curve of acaricides against eggs of* R. (B.) microplus*.

The effect of the different concentrations of cypermethrin, deltamethrin, fenvalerate, flumethrin, and amitraz was evaluated on the eggs of* R. (B.) microplus*. Results reveal that all concentrations of flumethrin and amitraz caused complete inhibition of hatching and zero hatching percentage was recorded. The hatching percentage was calculated as the number of hatched larvae divided by the total number of incubated eggs. This ratio integrates both mortality of eggs and the inability of viable eggs to hatch. Further, in case of other SPs (cypermethrin, deltamethrin, and fenvalerate), an increasing trend in the mortality of viable tick eggs and thus a decrease in the hatching percentage were recorded when the concentration of acaricides increased. A hatchability of 31.0 ± 6.1, 40.0 ± 5.2, and 19.3 ± 1.7% was recorded at the highest concentrations of cypermethrin, deltamethrin, and fenvalerate, respectively. The regression graph of probit hatchability of eggs plotted against log values of progressively increasing concentrations of various acaricides is shown in Figure 1. The dotted lines in the regression curve represented the 95% confidence limits. The slope of hatchability (95% confidence limits), Y-intercept (95% CL), the value of goodness of fit* (R* ^*2*^ *),* and* P* values of various acaricides used against the eggs of* R. (B.) microplus* are shown in Table 1. The increase in the concentration of the acaricide showed a significant effect on the hatchability of eggs for cypermethrin (*P* < 0.01) and deltamethrin (*P* < 0.05) but was nonsignificant for fenvalerate.

**Figure 2:**
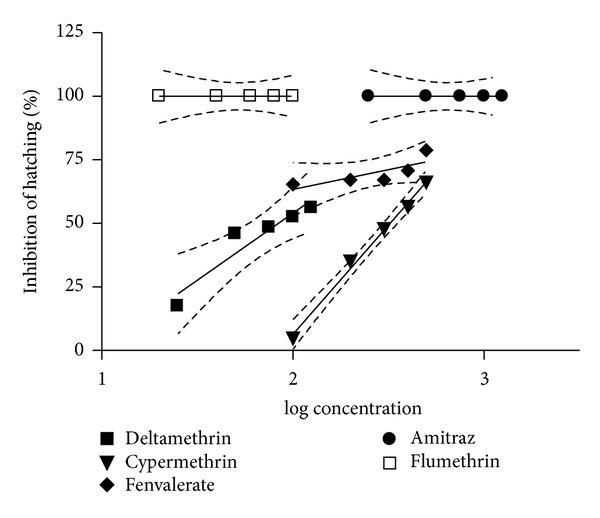
Dose-IH (%) curve of acaricides against eggs of* R. (B.) microplus.*

The dose response curves of eggs of* R. (B.) microplus *against log values of progressively increasing concentrations of various acaricides were plotted for percentage inhibition of hatching IH% by the data generated by EHA (Figure 2). As there was an increase in the IH% in eggs with increase in drug concentration, thus, a positive slope was recorded for various acaricides. Similar to hatching (%) an increase in the concentration of the acaricide showed a significant effect on the IH% of eggs for cypermethrin (*P* < 0.01) and deltamethrin (*P* < 0.05) but was nonsignificant for fenvalerate. The slope of the regression curve of IH% was utilized for the estimation of the dose of various acaricides causing inhibition of hatching for 95% eggs (LC_95_) and the discriminating dose (DD). The slope of IH% (95% CL), Y-intercept (95% CL), the value of* R* ^*2*^,* P* values, LC_95_, and DD of various acaricides used against the eggs of* R. (B.) microplus* are shown in Table 2. Results indicate that maximum DD was recorded for fenvalerate (2.136%), followed by cypermethrin (0.214%) and deltamethrin (0.118%).

Control of cattle tick* R. (B.) microplus* rests on continuous use of acaricides on and off the hosts. Long-term use of these chemicals is leading to the development of resistance, issues around the residues in livestock products and in environment, and their undesirable effects [15]. Among the various acaricides used in India for the control of ticks in livestock, resistance development was first reported against *γ*BHC in* R. (B.) microplus* [16, 17] followed by dieldrin [18], sevin [19], lindane [20], diazinon [6], synthetic pyrethroids [2, 21–23], amitraz [24, 25], and malathion [26]. However, in the current system of livestock production in developing countries, the tick control cannot be imagined without the use of acaricide despite the increasing resistant tick population due to the absence of newer generation acaricides.

As per a recent study by Sharma et al. [2] the LC_95_ values of cypermethrin and deltamethrin by adult immersion test against susceptible IVRI-I line of* R. (B.) microplus* were reported as 349.1 and 29.6 ppm with DD of 698.2 ppm and 59.2 ppm, respectively. Similarly, in larval packet test, the LC_95_ values of cypermethrin and deltamethrin were 350.7 ppm and 35.5 ppm, respectively, against larvae of susceptible IVRI-I line of* R. (B.) microplus*. However, the results of current study demonstrate that much higher concentration of cypermethrin and deltamethrin would be required for the efficient control of the egg stage, thus indicating that the dose at which these acaricides are being used in field conditions is ineffective and needs to be revalidated. Further, in the current study, commercially available acaricides were used to assess the efficacy of these widely used drugs which could not have been possible with the use of analytical grade acaricides as commercial products are prepared with many proprietary ingredients and it is difficult to assess the responses due to individual components of the formulations.

However, it has been proposed that intermittent use of high concentration of acaricides to kill ticks with resistant alleles may provide a basic means of delaying resistance [27]. Increased concentration of acaricide has been used successfully in controlling DDT-, OP-, and SP-resistant strains of* R. (B.) microplus *[28]. Still this strategy is not widely used as potential host toxicity and chemical residue problems now need to be reconsidered before an increased concentration could be used for resistance management. Although the results of current study indicate that a much higher concentration of these acaricides is required for the destruction of egg stages of the tick off the host, the same can be applied at a much higher dose in the shed without risking the host toxicity.

The results of the current study reveal a state of ineffectiveness against the egg stage of* R. (B.) microplus* of these commonly used SP acaricides, whereas other acaricides (amitraz and flumethrin) showed cent per cent efficacy against these eggs and hence can be better option for the effective control of egg stages of ticks mainly off the host at their breeding ground particularly for the treatment of the sheds. The data generated would be useful in effective control of tick stages off the host and would hence lead to lesser number of infective stages available in the environment and would thus contribute immensely to control of ticks.

## Figures and Tables

**Table 1:** The results of egg hatch assay to various acaricides on eggs of *R. *(*B.*)* microplus*.

Acaricide	Slope of hatchability (95% CL)	*Y*-intercept (95% CL)	*R* ^ 2^	*P* value
Amitraz	—	—	—	—
Cypermethrin	−2.266 ± 0.1671 (−2.798 to −1.734)	10.59 ± 0.4059 (9.298 to 11.88)	0.9839	0.0009^a^
Deltamethrin	−1.260 ± 0.2620 (−2.094 to −0.4266)	7.311 ± 0.4795 (5.786 to 8.837)	0.8852	0.0171^b^
Fenvalerate	−0.4578 ± 0.2208 (−1.160 to 0.2447)	5.506 ± 0.5361 (3.800 to 7.212)	0.5890	0.1298
Flumethrin	—	—	—	—

^a^(*P* < 0.01); ^b^(*P* < 0.05).

**Table 2:** Dose-percent inhibition of hatching of various acaricides to *R. *(*B.*)* microplus*.

Acaricide	Slope (95% CL)	*Y*-intercept (95% CL)	*R* ^ 2^	*P* value	LC_95_ (%)	DD (%)
Amitraz	—	—	—	—	NC	NC
Cypermethrin	85.97 ± 3.791 (73.91 to 98.04)	−165.6 ± 9.206 (−194.9 to −136.3)	0.9942	0.0002^a^	0.107	0.214
Deltamethrin	52.83 ± 10.24 (20.24 to 85.43)	−51.65 ± 18.74 (−111.3 to 7.998)	0.8987	0.0141^b^	0.059	0.118
Fenvalerate	15.58 ± 6.848 (−6.207 to 37.37)	32.21 ± 16.63 (−20.71 to 85.12)	0.6329	0.1075	1.068	2.136
Flumethrin	—	—	—	—	NC	NC

NC: could not be calculated as 100% inhibition was recorded at all concentrations tested.

^a^(*P* < 0.01); ^b^(*P* < 0.05).
